# Evaluation of incision healing status after transverse uterine fundal incision for cesarean delivery and postoperative pregnancy: a ten-year single-center retrospective study

**DOI:** 10.1186/s12884-024-06446-7

**Published:** 2024-04-15

**Authors:** Fumikazu Kotsuji, Takashi Shibata, Satoshi Nakago, Hiroki Kato, Sayoko Hosono, Yasunori Fukuoka, Koji Nishijima

**Affiliations:** 1 Department of Obstetrics and Gynecology, Takatsuki General Hospital, Takatsuki, Japan; 2 Center for Perinatal, Maternal and Neonatal Medicine, Niigata University Medical and Dental Hospital, Niigata, Japan

**Keywords:** Cesarean section, Postoperative pregnancy, Obstetrics, Transverse uterine fundal incision, Uterine rupture, Wound scar

## Abstract

**Background:**

Transverse uterine fundal incision (TUFI) is a beneficial procedure for mothers and babies at risk due to placenta previa-accreta, and has been implemented worldwide. However, the risk of uterine rupture during a subsequent pregnancy remains unclear. We therefore evaluated the TUFI wound scar to determine the approval criteria for pregnancy after this surgery.

**Methods:**

Between April 2012 and August 2022, we performed TUFI on 150 women. Among 132 of the 150 women whose uteruses were preserved after TUFI, 84 women wished to conceive again. The wound healing status, scar thickness, and resumption of blood flow were evaluated in these women by magnetic resonance imaging (MRI) and sonohysterogram at 12 months postoperatively. Furthermore, TUFI scars were directly observed during the Cesarean sections in women who subsequently conceived.

**Results:**

Twelve women were lost to follow-up and one conceived before the evaluation, therefore 71 cases were analyzed. MRI scans revealed that the “scar thickness”, the thinnest part of the scar compared with the normal surrounding area, was ≥ 50% in all cases. The TUFI scars were enhanced in dynamic contrast-enhanced MRI except for four women. However, the scar thickness in these four patients was greater than 80%. Twenty-three of the 71 women conceived after TUFI and delivered live babies without notable problems until August 2022. Their MRI scans before pregnancy revealed scar thicknesses of 50–69% in two cases and ≥ 70% in the remaining 21 cases. And resumption of blood flow was confirmed in all patients except two cases whose scar thickness ≥ 90%. No evidence of scar healing failure was detected at subsequent Cesarean sections, but partial thinning was found in two patients whose scar thicknesses were 50–69%. In one woman who conceived seven months after TUFI and before the evaluation, uterine rupture occurred at 26 weeks of gestation.

**Conclusions:**

Certain criteria, including an appropriate suture method, delayed conception for at least 12 months, evaluation of the TUFI scar at 12 months postoperatively, and cautious postoperative management, must all be met in order to approve a post-TUFI pregnancy. Possible scar condition criteria for permitting a subsequent pregnancy could include the scar thickness being ≥ 70% of the surrounding area on MRI scans, at least partially resumed blood flow, and no abnormalities on the sonohysterogram.

**Trial registration:**

Retrospectively registered.

## Background

Placenta previa-accreta, a major cause of maternal mortality by massive hemorrhage, has increased in incidence, particularly due to the growing rate of repeated Cesarean sections (CS) [1–4]. It is difficult to avoid transecting the placenta by traditional low-transverse CS, especially when the placenta covers the entire anterior uterine wall in this abnormal placentation. This often results in catastrophic hemorrhage and fetal and maternal anemia. The use of a vertical uterine incision has been reported [5], but transecting the placenta is unavoidable when the placenta broadly involves the anterior uterine wall. We have developed the CS with transverse uterine fundal incision (TUFI) technique to avoid transecting the placenta in such cases [6]. An incision into the placenta can be completely avoided with TUFI. In addition, bleeding is minimal because the muscle layer of the uterine fundus is thinner than the uterine body [7] and the incision is made parallel to the path of the arcuate artery [8]. TUFI is also characterized by strong uterine contractions after placental delivery [7, 9], which suppress bleeding from the detached plane of the placenta without the need for additional compression hemostatic methods. Due to its advantages of safety for the mother and neonate and ease of performance for the surgeon, TUFI is now widely used as an operative method for placenta previa-accreta [10]. TUFI has also been useful in *en caul* delivery of extremely low birth-weight infants [9].

Even with placenta previa-accreta, the uterus can be preserved after TUFI when the degree of villous invasion into the myometrium is mild or the area of invasion is not extensive. Some patients can therefore wish to conceive again, although TUFI was originally developed as a last resort to save the lives of mothers and fetuses without considering a subsequent pregnancy. The risk of uterine rupture during a subsequent pregnancy after TUFI remains unclear, while methods of predicting uterine rupture during a subsequent pregnancy after traditional low-transverse incisions have been reported [11]. Based on our ten years of experience, we report here the wound healing status and subsequent pregnancies after TUFI, and also discuss possible criteria for approving a subsequent pregnancy after TUFI.

## Methods

To determine the approval criteria for pregnancy after TUFI, we retrospectively evaluated the uterine scars in patients after this operative method. This retrospective study was conducted with the approval of the Takatsuki General Hospital Ethics Committee (permission number: 2021-40). Informed consent was obtained via an opt-out system on the website.

### Patients

**Fig. 1:**
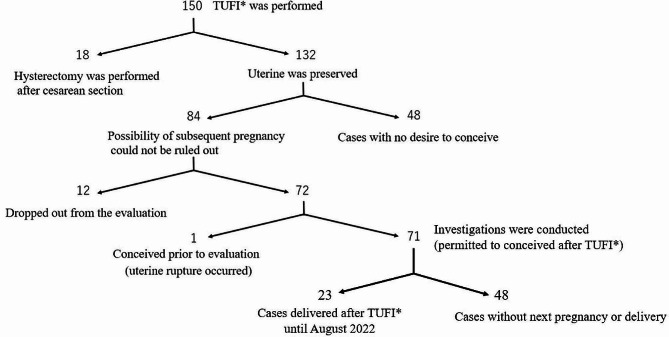
Flowchart of patients after transverse uterine fundal incision for Cesarean delivery TUFI*: transverse uterine fundal incision for Cesarean delivery

Between April 2012 and August 2022, we performed TUFI on 150 women at our hospital. Eighteen cases resulted in hysterectomy, 17 of which were due to placenta accreta, which invades the scar of the previous CS or myomectomy, while the remaining case had massive bleeding caused by retained products of conception one week after TUFI. The uterus was preserved post-TUFI in 132 women. Of these, 84 women wished to conceive again after TUFI and were enrolled in the study (Fig. 1). All participants whose data was shown in this manuscript gave informed consent to the reporting of their details. Patient anonymity has been preserved.

### Closure method for TUFI

**Fig. 2:**
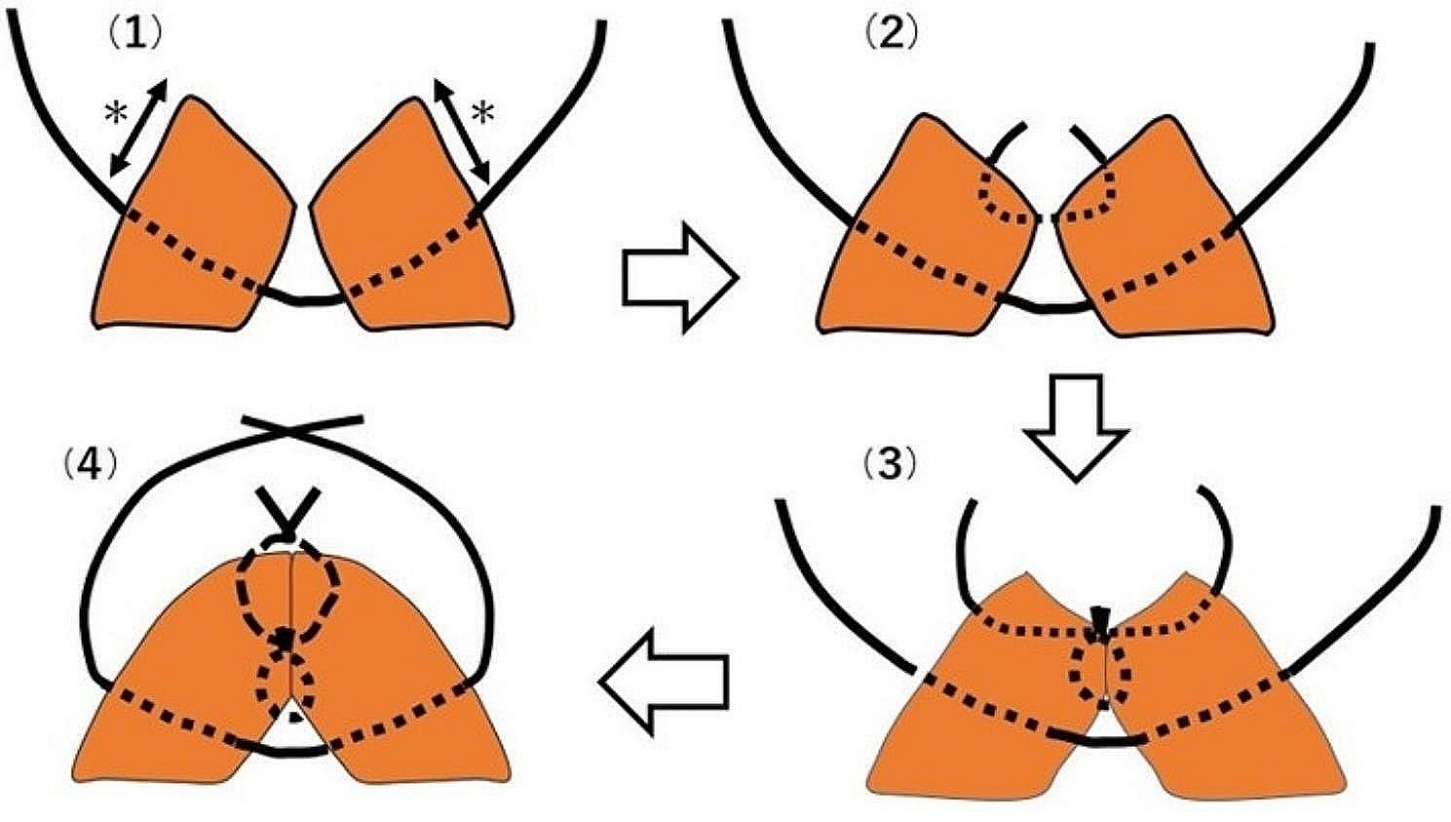
The closure method for transverse uterine fundal incision Interrupted retention sutures were set as wide as possible, as indicated by asterisk (**1**). Myometrial wound was closed with two layers of interrupted sutures (**2** and **3**). The retention sutures were then tied (**4**). Absorbable 1 − 0 and 2 − 0 threads were used for the retention suture and myometrial wound closure, respectively

We recommend the closure method with added interrupted retention sutures for TUFI to prevent dehiscence of the uterine wound, which is caused by strong uterine contractions during puerperium. Briefly, five interrupted retention sutures (absorbable 1 − 0 threads) were set as wide as possible, and the myometrial wound was closed using two layers of interrupted sutures (absorbable 2 − 0 threads). The retention sutures were then tied [6, 12] (Fig. 2).

### Evaluation of the post-TUFI scars

The scar formation status was evaluated by magnetic resonance imaging (MRI) and sonohysterogram 12 months after TUFI. We used 3.0 Tesla MRI (MAGNETOM Skyra, Siemens, Germany) and the imaged sections were 3 mm thick. Four surgeons evaluated MRI images independently, and the value with the strongest indentations and smallest range of enhancement was adopted, excluding the surgeon who performed the TUFI procedure. In addition, one of the four examiners performed the sonohysterographic evaluation. Further details are explained below.

#### Evaluation of TUFI scar thickness by MRI

**Fig. 3:**
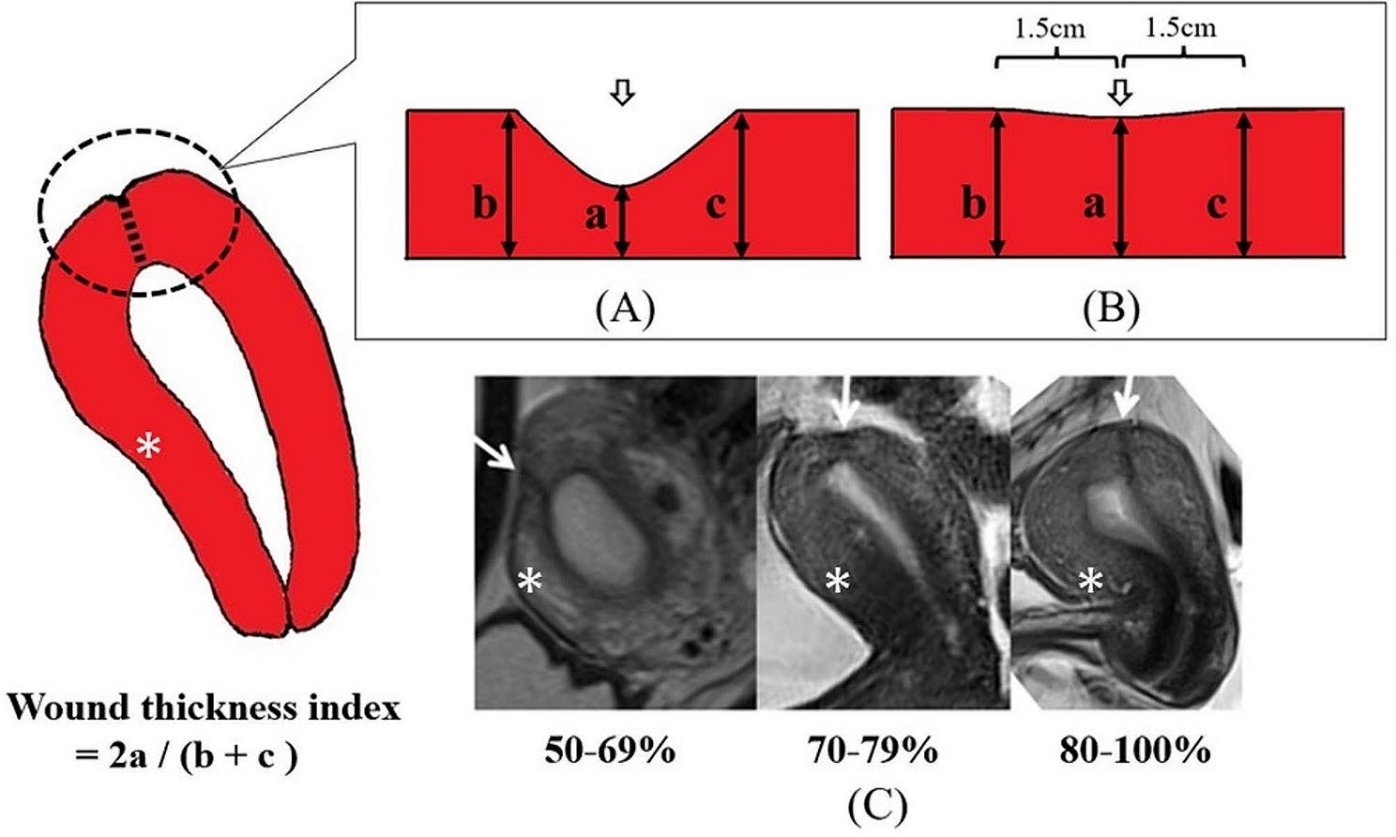
MRI evaluation of the scar thickness 12 months after transverse uterine fundal incision (**A**) The thickness of the thinnest part of the scar (a) was compared with the thickness of both ends of the scar (b and c), and the scar thickness was assessed using the “wound thickness index” expressed as 2a/ (b + c) (**B**) In cases where the scar ends were unclear, the points located 1.5 cm away from the thinnest part were designated as “b” and “c” of the wound thickness index (**C**) Sample images of the “wound thickness index” Arrow: the thinnest part of transverse uterine fundal incision scar Asterisk: anterior wall of the uterus

The TUFI scar thickness was evaluated using T2-weighted images in the sagittal view. Evaluating the scar formation by measuring only the thinnest part of the scar can be unreliable, since there are individual differences in the thickness of the muscular layer of the non-pregnant uterine wall. In one report, scar formation was evaluated using the ratio of the residual myometrium to the adjacent myometrium on one side [13]. In our study, to make the evaluation more objective, the thickness of the thinnest part of the scar (“a” in Fig. 3) was compared with the thickness of both ends of the wound (“b & c” in Fig. 3). We assessed the scar thickness using the “wound thickness index” expressed as *2a / (b + c)* (Fig. 3). When the indentation of the scar compared to the surrounding muscle layer was slight, the scar ends were unclear. In such cases, we set points b and c at 1.5 cm away from the thinnest part (“(B)” in Fig. 3).

#### Evaluation of vascularization in TUFI scar by MRI

**Fig. 4:**
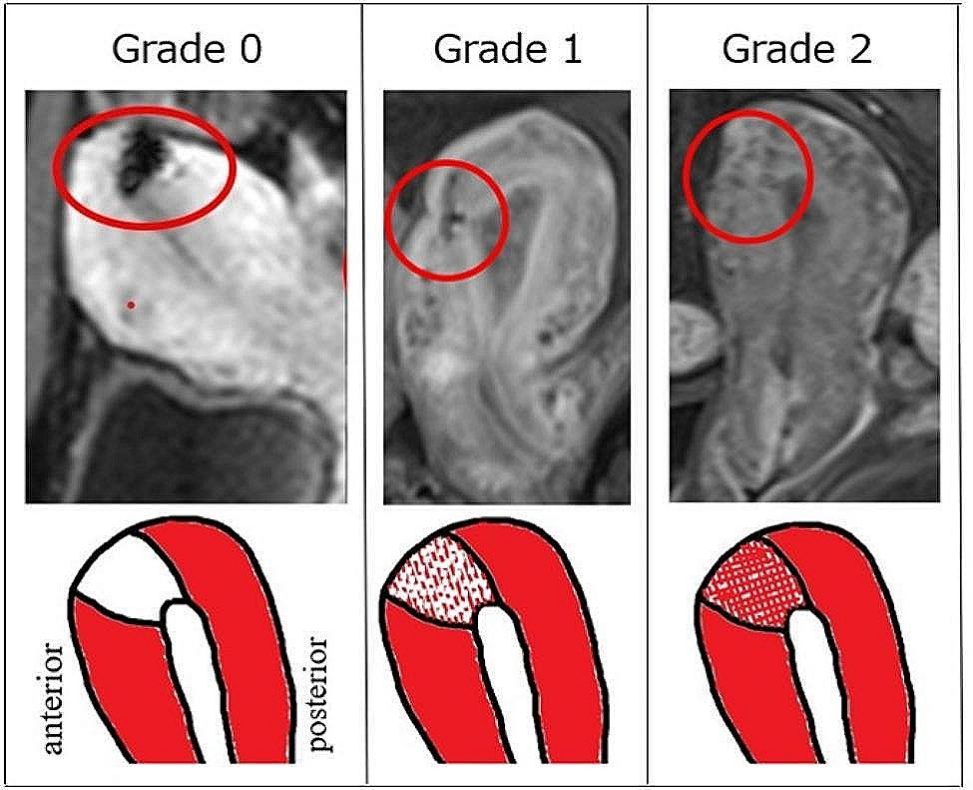
MRI evaluation of vascularization in scar 12 months after TUFI using dynamic contrast-enhanced images The percentage of enhanced area in the scar was visually evaluated and classified into three categories as follows: Grade 0: the scar area was not enhanced Grade 1: parts of the scar area were enhanced Grade 2: ≥80% of the scar area was enhanced

Vascularization of the TUFI scar was evaluated using dynamic contrast-enhanced images obtained with the T1-weighted sequence (Fig. 4). Angiogenesis is an important component in the remodeling of tissues, and its analysis is a standardized method for assessing vascular physiological characteristics [14, 15]. The percentage of the enhanced area in the scar was visually evaluated based on the subjective opinion of the evaluators. Grade 0 was assigned to cases in which the scar area was not enhanced, Grade 1 was assigned to cases in which parts of the scar area were enhanced, and Grade 2 was assigned to cases with enhancement of ≥ 80% of the scar area.

#### Examination by sonohysterogram

**Fig. 5:**
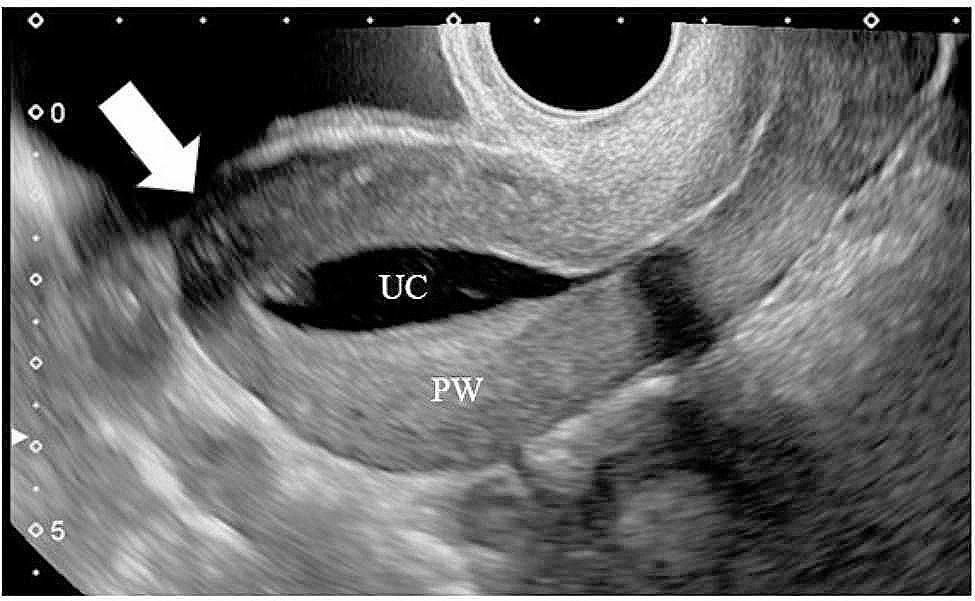
Examination of the scar 12 months after transverse uterine fundal incision by sonohysterogram While the uterine cavity was expanded with a saline injection, the scar area was observed by transvaginal ultrasonography Arrow: the transverse uterine fundal incision scar UC: uterine cavity filled with saline PW: posterior wall of the uterus

A sonohysterogram was performed to check the morphology of the scar when the uterine cavity was expanded with a saline injection. In brief, a 12 Fr catheter was inserted into the uterine cavity. During the saline injection, the TUFI scar site was then observed using transvaginal ultrasonography (Fig. 5).

#### Direct observation of the TUFI scar during CS at subsequent pregnancies

The TUFI scar was assessed immediately after placental delivery to confirm whether the MRI assessment of wound healing was consistent with the intraoperative findings. The scar thickness was examined and compared with the thickness of the surrounding muscular layer by visual and bimanual examination. If the placenta covered the TUFI scar, the presence of placenta-accreta was checked. Three surgeons evaluated the thickness of the scars, and the thinnest value was adopted. The surgeon who had performed the TUFI was excluded from being the operator of the subsequent CS.

### Tentative criteria for post-TUFI pregnancy approval during this study period

We approved subsequent pregnancies after TUFI for cases that satisfied both the following scar conditions and postoperative management. Regarding the scar conditions, we developed tentative and somewhat subjective criteria to approve a post-TUFI pregnancy based on the results of the postoperative MRI and sonohysterographic examinations. In principle, a post-TUFI pregnancy was approved when all of the following findings were present: MRI scans showed a wound thickness index of ≥ 50% and at least part of the scar was enhanced on dynamic contrast-enhanced MRI, and the sonohysterogram showed no abnormalities that had not been detected by MRI. We also provisionally approved a post-TUFI pregnancy in patients without enhancement of the scar but with a wound thickness index of ≥ 80%.

Regarding postoperative management, we urged women planning any future pregnancies to undergo an imaging test one year after TUFI and to obtain permission to become pregnant based on the results. Women who conceived post-TUFI were hospitalized after 25 weeks of gestation to prepare for the possibility of sudden uterine rupture [6]. Elective CS was scheduled between 34 and 37 weeks of gestation after fetal maturation and before the onset of labor. Patients were informed of the potential risk of uterine rupture and provided consent based on the above management protocol.

## Results

### Follow-up after TUFI

Twelve of the 84 patients dropped out from the post-TUFI evaluation of incision-healing status. One patient conceived seven months post-TUFI, prior to the evaluation of the post-TUFI scars, and uterine rupture occurred at 26 weeks of gestation (as will be discussed in detail below). Investigations were therefore conducted on the remaining 71 cases (Fig. 1).

### Incision-healing status twelve months after TUFI as evaluated by MRI and sonohysterogram

**Fig. 6:**
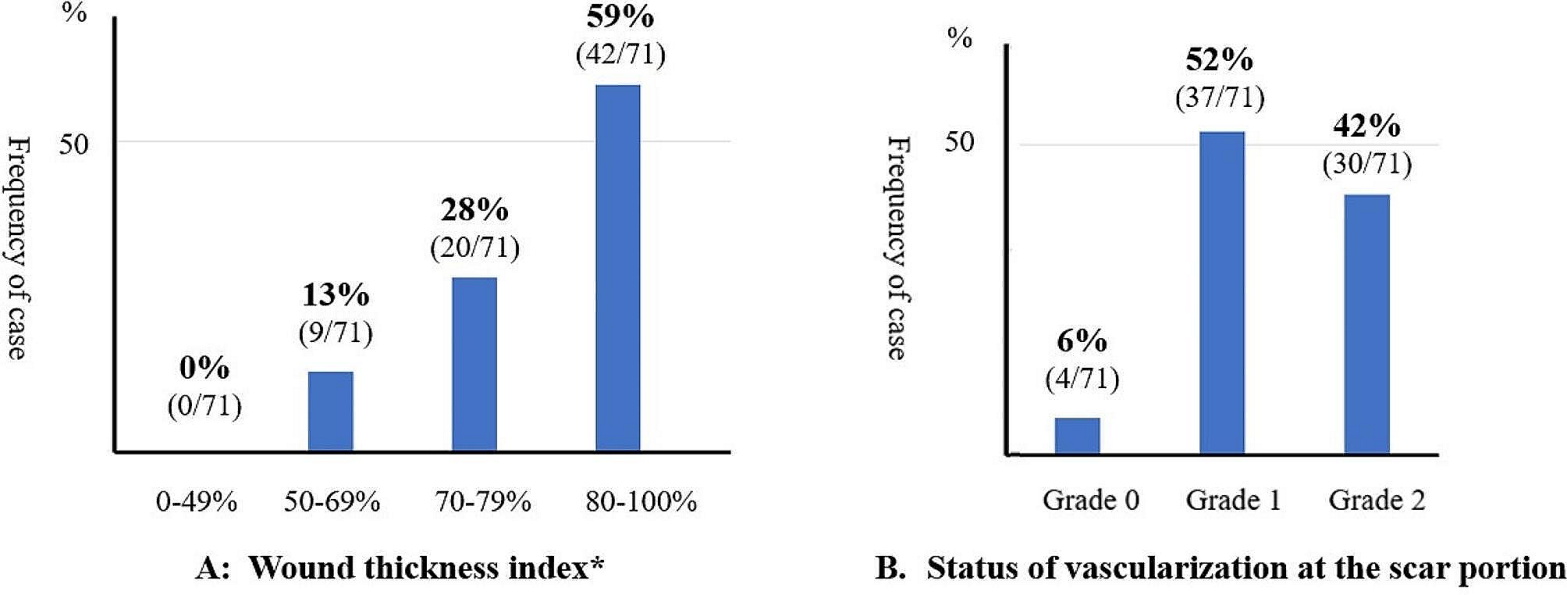
MRI evaluation of incision-healing status twelve months after transverse uterine fundal incision **A**: All the patients had a wound thickness index ≥ 50% and approximately 90% of the patients had an index ≥ 70% Wound thickness index*: The thickness of the scar compared with the thickness of the surrounding muscle layer by MRI **B**: Vascularization status Grade 0, 1, and 2 was found in 4 (6%), 37 (52%), and 30 (42%) women, respectively. The scar area of four patients was not enhanced based on dynamic contrast-enhanced images (Grade 0), but their wound thickness index was ≥ 80%

In all 71 women, the wound thickness index was ≥ 50% on their MRI scans: 50 ∼ 69%, 70 ∼ 79%, and > 80% in 9 (13%), 20 (28%), and 42 (59%) women, respectively (Fig. 6A). Vascularization status Grade 0, 1, and 2 was found in 4 (6%), 37 (52%), and 30 (42%) women, respectively (Fig. 6B). The scar area in four women was not enhanced, but their wound thickness index was > 80%. In addition, the sonohysterogram showed no abnormal findings that had not been detected by MRI in any of the cases.

### Post-TUFI conception (table 1)

**Table 1:** Details of twenty-three patients who conceived and delivered after transverse uterine fundal incision

Case No.	Data of TUFI^*1^ wound					Delivered twice post-TUFI^*1^	Surgical outcome			
	Incision position^*2^	MRI findings (12 months after TUFI^*1^)		Findings in Cesarean Section at the subsequent pregnancy			Previous Cesarean Section (TUFI^*1^)		Subsequent Cesarean Section	
		Wound thickness index^*3^	Vasculari-zation^*4^	Visual observation^*5^	Bimanual examination^*6^		Placenta-accreta (clinically)	Total fluid loss^*7^	Gestational age (weeks)	Total fluid loss^*7 *8^
1	posterior	100%	grade 0	A	25%	+		1643 g	34	2130 g
2	anterior	97%	grade 1	B	50%	+		1178 g	37	1633 g
3	posterior	96%	grade 2	A	75%			891 g	36	1275 g
4	posterior	96%	grade 2	A	75%			2209 g	34	3037 g
5	anterior	94%	grade 0	A	50%		+	2400 g	36	977 g
6	posterior	93%	grade 2	A	50%			1369 g	34	1708 g
7	anterior	92%	grade 2	A	75%			388 g	35	1011
8	posterior	91%	grade 1	C	50%			1820 g	36	1935
9	posterior	87%	grade 1	B	50%			1016 g	36	1209
10	posterior	86%	grade 2	A	50%			866 g	36	711 g
11	posterior	83%	grade 2	A	25%			521 g	36	705 g
12	posterior	83%	grade 1	B	50%			507 g	36	1065 g
13	posterior	81%	grade 1	A	50%			1130 g	36	1276 g
14	posterior	81%	grade 1	A	50%			575 g	33	732 g
15	anterior	79%	grade 1	A	25%			689 g	34	2207 g
16	anterior	77%	grade 2	C	75%			1830 g	37	1836 g
17	posterior	77%	grade 1	A	75%			495 g	36	1815 g
18	posterior	77%	grade 1	B	50%			680 g	36	2285 g
19	posterior	73%	grade 1	B	50%			864 g	36	1734 g
20	posterior	71%	grade 1	C	50%	+		724 g	36	909 g
21	anterior	70%	grade 2	A	50%			1099 g	36	933 g
22	posterior	69%	grade 1	C	25%			1377 g	34	1055 g
23	anterior	53%	grade 1	C	25%			874 g	36	1412 g

*1. TUFI: transverse uterine fundal incision for Cesarean delivery

*2. The incision was made several cm away either anterior or posterior to the line connecting the Fallopian tube angles on both sides

*3. The thickness of the scar area was compared with the thickness of the surrounding muscle layer by MRI (see Fig. 3)

*4. The visual assessment of the enhanced scar area using contrast-enhanced MRI was performed as follows: grade 0: not enhanced, grade 1: parts of the scar were enhanced, grade 2: >80% of the scar was enhanced (see Fig. 4)

**Fig. 7:**
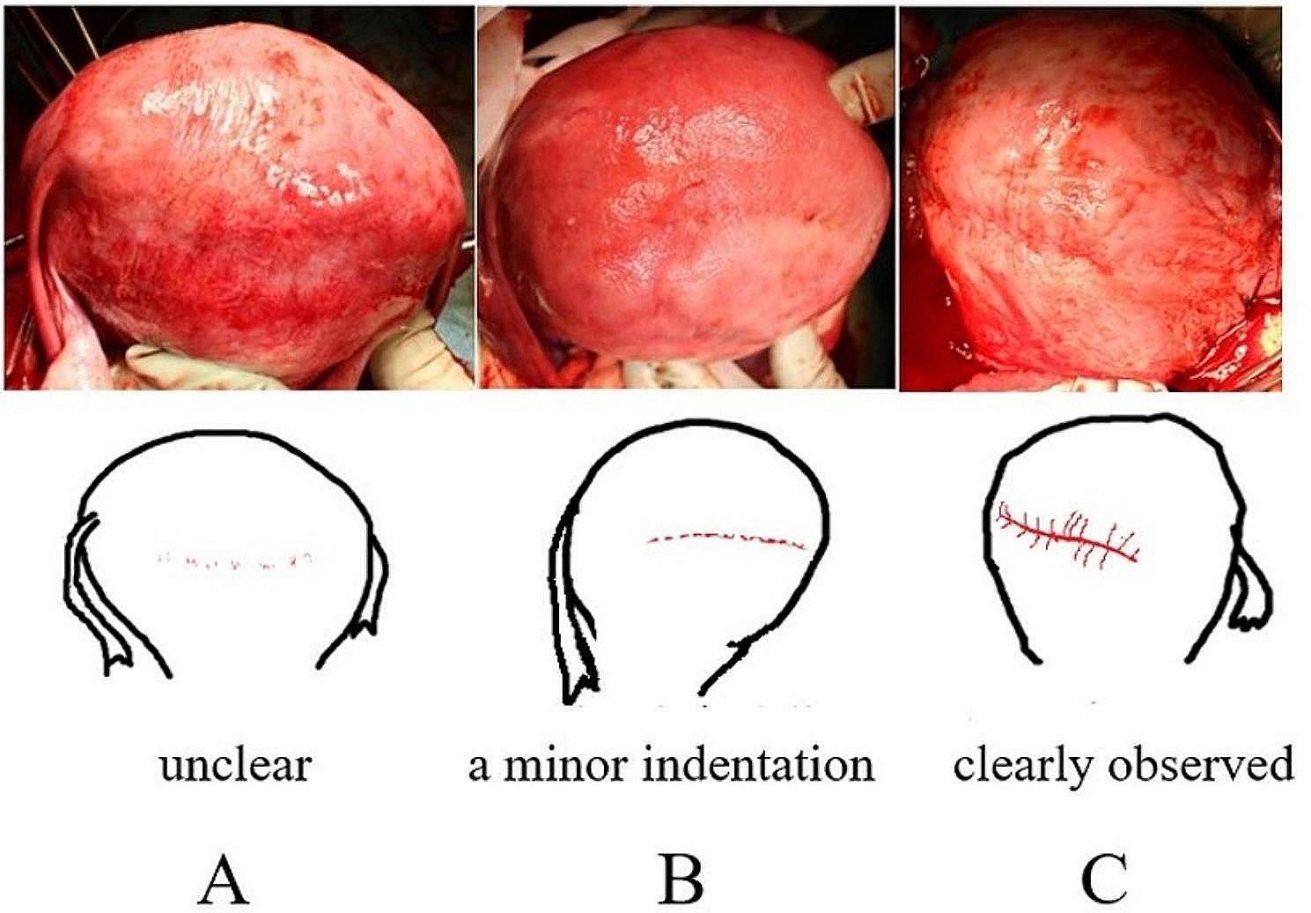
The TUFI scar observed after placental delivery during Cesarean section in the subsequent pregnancy **A**: An indentation was slightly discernible but difficult to find (unclear) in 11 patients. **B**: A minor indentation was observed in 7 patients. **C**: An indentation was clearly observed in 5 patients. **A** and **B** show the posterior wall and **C** shows the anterior wall of the uterus

*5. The scar area was visually examined during the Cesarean section in the subsequent pregnancy. A: the indentation was difficult to find, B: a minor indentation was observed, C: the indentation was clearly observed (see Fig. 7)

*6. The TUFI wound thickness was compared to the thickness of the surrounding normal tissue after placental delivery by bimanual examination during the Cesarean section in the subsequent pregnancy

*7. Total fluid loss included both blood and amniotic volumes

*8. Among four patients with total fluid loss of over 2000 g, two patients lost a large amount of amniotic fluid (cases 1 and 15), one patient had a myomectomy during surgery (case 18), and one patient had a hematologic medical history (case 4)

Based on the above results, all 71 women under investigation were approved to become pregnant. Twenty-three of the 71 women conceived after TUFI, and all of them delivered live babies with CS until August 2022. Of these, 19 delivered on schedule between 34 and 37 weeks of gestation, and four patients delivered earlier than scheduled between 33 and 35 weeks of gestation due to preeclampsia or the onset of labor.

#### MRI findings at twelve months after TUFI limited to the 23 women who conceived and delivered post-TUFI (Table 1

The wound thickness index of these 23 women, as evaluated by post-TUFI MRI, was 50–69% in 2 cases and ≥ 70% in the remaining 21 cases. The scar area was not enhanced on contrast-enhanced MRI in two women whose wound thickness index was ≥ 90%, but at least partial enhancement was detected in the remaining 21 women.

#### Findings of the TUFI scar during the CS in the subsequent pregnancy (Figs. 7 and 8 and Table 1)

**Fig. 8:**
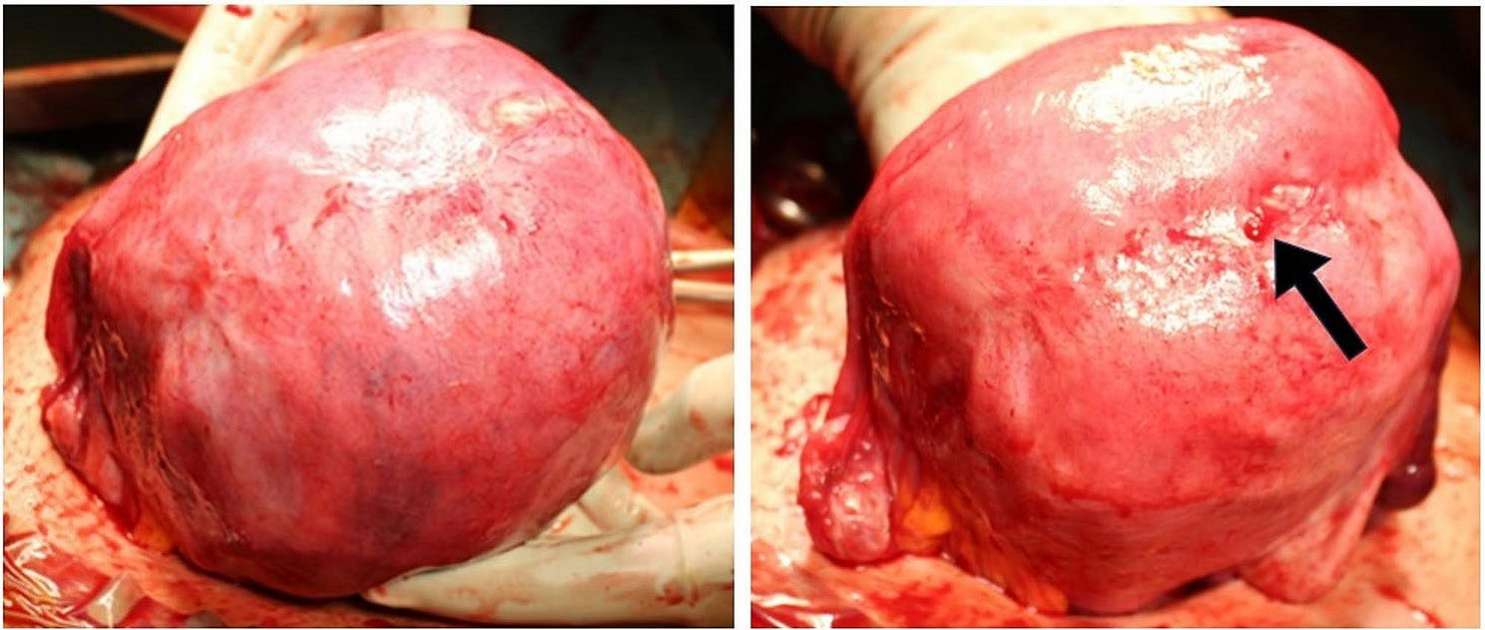
Partial scar thinning detected after placental delivery in cases with scar thicknesses of 50–69% TUFI scars were not noticeable before placental delivery (left) but became apparent after delivery (right) in cases with scar thicknesses of 50–69% Arrow: thinning part of the TUFI scar

TUFI scars observed during the CS of the subsequent pregnancy are shown in Fig. 7.

In these cases, TUFI scars which were not noticeable before placental delivery became apparent after delivery. In 11 women, an indentation was difficult to find (unclear, Fig. 7A). A minor indentation was observed in 7 women (Fig. 7B), and indentations were clearly observed in 5 women (Fig. 7C). With bimanual examination after placental delivery, the TUFI scar thickness was found to be approximately 25% of the thickness of the surrounding muscular layer in 6 women, approximately 50% of this thickness in 11 women, and approximately 75% of this thickness in 6 women. In two cases whose MRI scar thickness index was 50–69% (case numbers 22 and 23 in Table 1), partial scar thinning was observed during the subsequent CS (Fig. 8). We performed an excision and repair of the thinning part of their uterine scar.

The placenta was found to be overlying the TUFI scar in five women, but placenta-accreta was not detected in any of them. All the subsequent CS procedures were performed using traditional lower transverse incisions. There was no abnormal bleeding related to the previous TUFI scar, with an average total fluid loss of 1093 g including both blood and amniotic volumes.

### Clinical course of a woman who conceived seven months after TUFI prior to wound evaluation

One woman conceived seven months post-TUFI before the evaluation, and spontaneous uterine rupture occurred at 26 weeks of gestation. During an emergency laparotomy, part of the chorioamniotic membrane was exposed to the abdominal cavity but both the fetus and placenta were present in the uterus. The patient lost 2550 g of blood including amniotic fluid. The child’s Apgar scores were 1 (1 min) and 2 (5 min) after birth.

At her previous CS (TUFI), total fluid loss was 1013 g including blood and amniotic volumes, no blood transfusion was required, and there were no postoperative infections or complications. Placenta-accreta was not confirmed during the previous surgery. Threatened preterm labor and premature rupture of membranes were not diagnosed before uterine rupture in the post-TUFI pregnancy.

## Discussion

Our findings from the TUFI scar evaluation and experience of post-TUFI pregnancies suggest that pregnancy after TUFI would not be contraindicated if certain conditions could be met. An appropriate suture method, contraception for at least 12 months, postoperative evaluation of TUFI scar formation status at 12 months, and cautious postoperative management, including patient education, are essential. In addition, the following criteria may need to be fulfilled to gain approval for a post-TUFI pregnancy: MRI scans showing a wound thickness index of ≥ 70% and at least partial enhancement of the scar, and the sonohysterogram showing no abnormalities that were undetected by MRI. All the patients in this study who met the above conditions delivered live babies without notable complications, and there were no abnormal findings during the subsequent CS in their pregnancies. Two patients with a scar thickness index of 50–69% delivered live babies after uneventful pregnancies, but there was partial scar thinning which left some concerns. Therefore, we concluded that it was better to adopt a wound thickness index of > 70% as approval criteria for a subsequent pregnancy.

**Fig. 9:**
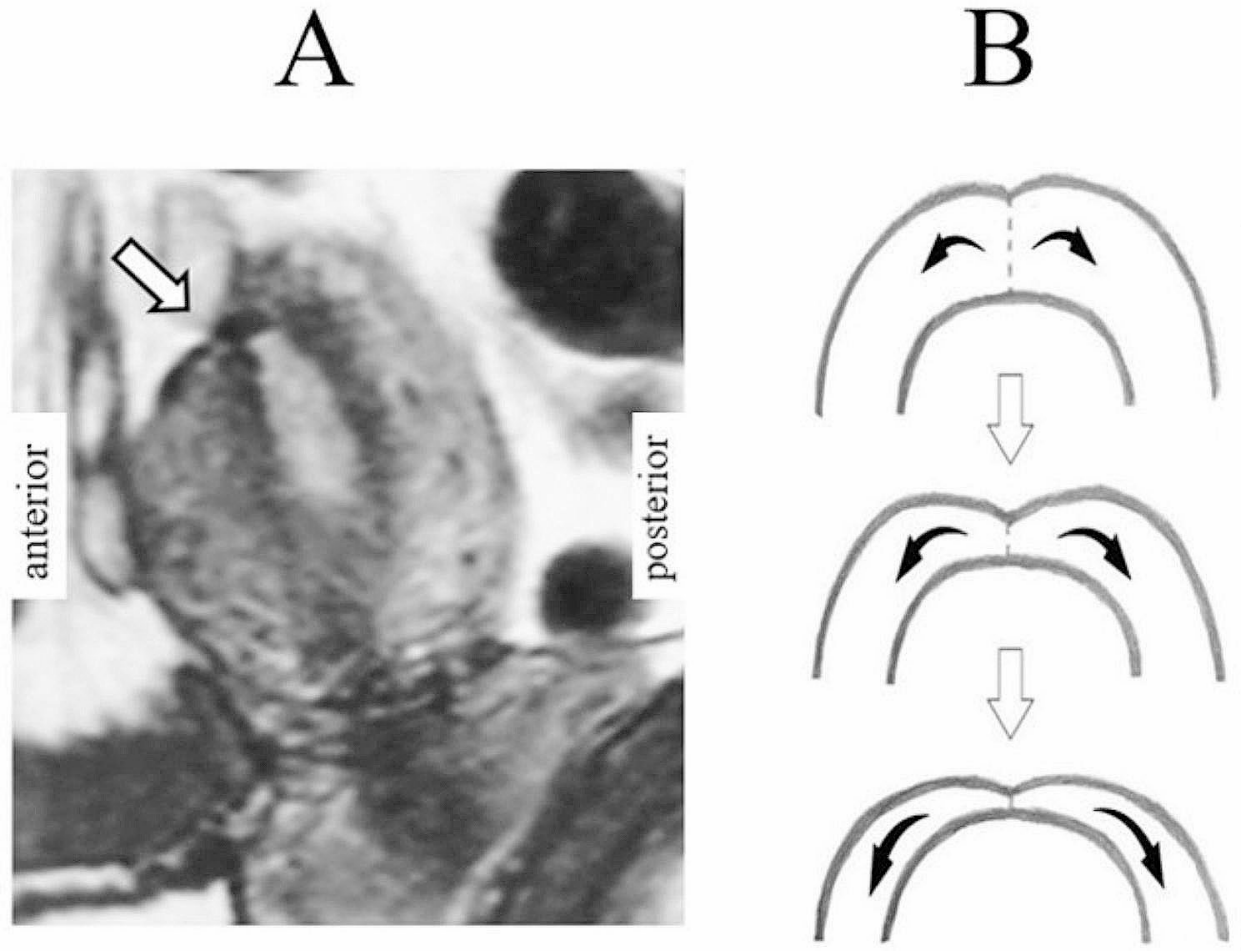
Wound healing status when closed by a single layer of interrupted sutures **A**: The wound thickness in MRI images after transverse uterine fundus incision in the early stages of TUFI development that was ≤ 30% of the thickness of the surrounding muscle layer Arrow: the scar from the transverse uterine fundal incision **B**: Schematic drawings indicate that the uterine muscle was pulled in both directions, suggesting that strong postpartum uterine contractions prevented wound healing

Studies investigating the association between the thickness of the CS scar at the lower uterine segment and uterine rupture during subsequent pregnancies have shown that the risk of uterine rupture increases as the thickness decreases [16–19]. Wound healing status can be improved by devising a more effective wound closure method to resist the effect of postpartum uterine contractions. When the uterine incision was closed by a single layer of interrupted sutures in the early stages of TUFI development, the wound thickness index was ≤ 30% in 16.7% of cases (Fig. 9A). The uterine muscle was assumed to be pulled in both directions by strong postpartum uterine contractions that prevented TUFI wound healing (Fig. 9B). To counter the effect of these strong postpartum uterine contractions, we changed the method of wound closure (Fig. 2). After the alterations in protocol, approximately 90% of women had a wound thickness index of ≥ 70%, and there were no cases with an index of < 50% in the present study. At examinations during the subsequent CS, the scar thickness measured after placental delivery using bimanual examination was approximately 25–75%, and was sometimes inconsistent with the preoperative MRI findings. In these cases, the scar thickness may have been evaluated as being much thinner by bimanual examination. This is because the scars did not have the same contractile function as the standard muscle layer, but were compared to a normal muscular layer that was thickened due to postpartum contractions.

The condition of the CS scar is strongly associated with uterine rupture during subsequent pregnancies [20]. Scar healing is generally known to progress over time, so the time period between the incision and the subsequent pregnancy is important [21]. In the case of a transverse incision in the lower uterine segment, six months was reported to be necessary for complete scar formation after CS [22]. The risk of uterine rupture during labor after CS tripled when the time period from the previous CS to the subsequent delivery was < 18 months, compared with a time period > 18 months (if expressed in terms of the period from CS to the establishment of the next pregnancy, this would be eight months) [21]. After a uterine body incision, however, the risk of uterine rupture during a subsequent pregnancy is higher than that after a lower uterine segment incision [23, 24]. We therefore recommend postoperative conception with a delay of at least 12 months after TUFI, which is double the time required for healing of the lower uterine segment incision (if expressed in terms of the interdelivery interval, this would be more than approximately 22 months).

In our study, spontaneous uterine rupture occurred in one woman who conceived prior to the evaluation. To our knowledge, this is the fourth reported case of uterine rupture that occurred during a post-TUFI pregnancy. One possible reason that may explain the uterine rupture in our patient is that the time period between TUFI and the subsequent pregnancy was too short and the strength of the TUFI scar was perhaps insufficient. The time period in this patient was much shorter (7 months) than that of the other 23 patients (12–89 months, average of 32 months) in our study.

As to other reported cases of uterine rupture after TUFI, one patient was diagnosed at 21 weeks of gestation and her wound was closed by only a single layer of interrupted sutures [12]. In another case, the TUFI scar was not evaluated before pregnancy, and the rupture occurred at 11 weeks of gestation [25]. An additional patient experienced uterine rupture at 33 weeks of gestation [26]. This patient conceived 5 years after TUFI, but there were no retention sutures. Also, the severe scar defect was revealed by MRI 12 months after TUFI. Despite the patient being forbidden to become pregnant considering the risk of uterine rupture, she hid it and conceived by in vitro fertilization at another clinic. Our recommended surgical procedures and postoperative management protocol were not followed in any of these reported cases.

The current study has several limitations. This is a study based on our experience at a single institution. The risks associated with subsequent pregnancy for patients without scar enhancement on contrast-enhanced MRI remain unclear. Conclusions about the necessity of hospitalization during a post-TUFI pregnancy have also not been reached. The number of post-TUFI pregnancies is insufficient to firmly determine the predictors for uterine rupture during post-TUFI gestation. Thus, further case studies and collaborative investigations are needed.

## Conclusion

To summarize, patients who require TUFI do not need to avoid this beneficial operative method because of their desire to conceive again. In order to approve a post-TUFI pregnancy, we recommend that an appropriate suture method, delay in conception for at least 12 months with evaluation of the TUFI scar, and cautious postoperative management be implemented. The following criteria for the scar conditions could ensure a safer subsequent pregnancy: wound thickness index of ≥ 70%, at least partially resumed blood flow on contrast-enhanced MRI, and no abnormalities on the sonohysterogram. Although further investigations are required to establish criteria for permitting post-TUFI conception, we believe that our findings could be informative for obstetricians who perform this operative method and patients who wish to conceive after TUFI.

## Data Availability

All data generated or analyzed during this study are included in this published article.

## Abbreviations

CS: Cesarean section(s)
MRI: Magnetic resonance imaging
TUFI: Transverse uterine fundal incision
